# A Bioactivity *Versus* Ethnobotanical Survey of Medicinal Plants from Nigeria, West Africa

**DOI:** 10.1007/s13659-014-0005-7

**Published:** 2014-03-02

**Authors:** Lydia L. Lifongo, Conrad V. Simoben, Fidele Ntie-Kang, Smith B. Babiaka, Philip N. Judson

**Affiliations:** 1 Chemical and Bioactivity Information Centre, Department of Chemistry, Faculty of Science, University of Buea, Buea, Cameroon; 2 Chemical and Bioactivity Information Centre, 22-23 Blenheim Terrace, Woodhouse Lane, Leeds, LS2 9HD UK; 3 Chemical and Bioactivity Information Centre, Granary Wharf House, 2 Canal Wharf, Leeds, LS11 5PY UK

**Keywords:** African traditional medicine, Bioactivity, Ethnobotany, Medicinal plants, Nigeria

## Abstract

Traditional medicinal practices play a key role in health care systems in countries with developing economies. The aim of this survey was to validate the use of traditional medicine within local Nigerian communities. In this review, we examine the ethnobotanical uses of selected plant species from the Nigerian flora and attempt to correlate the activities of the isolated bioactive principles with known uses of the plant species in African traditional medicine. Thirty-three (33) plant species were identified and about 100 out of the 120 compounds identified with these plants matched with the ethnobotanical uses of the plants.

## Introduction

It is estimated that 66–85 % of the world’s population depends directly on plants as medicines [1–3]. Since the existence of human civilization, plants and their by-products have been used by a large proportion of the population living in rural and urban areas for various purposes such as medicine, healthcare, food, clothing, shelter, agriculture, agrochemicals, pharmaceuticals, narcotics, etc. The sum total of this is referred to as ethnobotany [4]. Medicinal plants are defined as plants having one or more organs containing substances that can be used for therapeutic purposes or which are precursors for the synthesis of useful drugs [5]. Thus, medicinal plants represent, for the local population, a possibility of simple and cheap treatment. In addition, they represent sources of potentially important new pharmaceutical substances since all parts of a plant, from roots to seed heads and flowers, are employed in traditional remedies and can therefore act as sources of lead compounds [6]. More than 80 % of the world’s populations, especially in developing countries, depend on traditional systems of medicine for the treatment of a variety of diseases. This observation could be attributed to two main factors; inaccessibility of modern drugs and the low purchasing power within the populations living in the rural areas [7]. Moreover, some of the local remedies work and so the populations have no need for anything different.

From antiquity, mankind has been developing a traditional medicinal system, based on the knowledge of medicinal plants throughout the world [8]. Africans, in particular, have used medicinal plants and animal-derived remedies in their struggle for survival and in their quest for religious experiences. The World Health Organization (WHO) defines traditional medicine as practices, knowledge and belief systems which use minerals, plants and animal based remedies, spiritual therapies and exercises to prevent, treat and maintain wellbeing [9]. This knowledge became enriched over numerous generations due to experimentation but also through observations of animal behaviour [10]. Most often, knowledge of traditional medicine is only inherited orally, thereby facing the danger of being lost in favor of Western medicine (WM). Many Africans believe in the manifestation of life forces or spirits in every creation, and that these spirits constitute the heart of all life forms, natural events or non-living things. This gives herbal medicine a vital role in health care delivery systems especially in remote areas where clinics and hospitals are sparsely located [8, 11]. Despite the advances in WM, African traditional medicine (ATM) has gained renewed interest in the health care services throughout the continent. This could probably be due to the rapidly increasing awareness of the potential and curative abilities of alternative medicines, especially from the use of medicinal plants, as well as the inadequate access to WM and physicians and the high cost for Western drugs [12]. The argument for the local African populations resorting to traditional remedies could also be partly justified by the fact that natural product inspired molecules represented about 80 % of drugs that had been put into the market [1, 13].

Previous ethnobotanical studies of medicinal plants confirm the rational use of recipes by different people or groups from different communities [14]. Numerous varieties of medicinal plants growing in Africa are widely used against many diseases ranging from endemic tropical diseases like malaria [15] and leishmaniasis [16] to complex illnesses such as asthma [17], psychosis [18], hepatitis [19] and even cancer [20]. Searching for new lead compounds to be developed as drugs or as templates for analogue synthesis and the evaluation of traditional medicine and herbal medicinal products, are the two basic reasons for the advancement of work on medicinal plants [21]. The use of plant preparations can be supported if it is safe and if their biological activity can be scientifically confirmed. This calls for quality control and standardization. If the activity cannot be confirmed, and certainly if there is a risk of toxicity, the use of herbal medicinal products should be discouraged [21]. The study of plants used traditionally as medicines is therefore an interesting discipline because of the possibility to find new drugs and also because of the strong adhesion of local populations, for the aforementioned reasons [11–13, 22, 23]. This has prompted many research teams to carry out studies on plants used in Africa by traditional healers against diseases.

The United Nations definition of Western Africa includes the following 16 countries: Benin, Burkina Faso, Cape Verde, Ivory Coast, the Gambia, Ghana, Guinea, Guinea-Bissau, Liberia, Mali, Mauritania, Niger, Nigeria, Senegal, Sierra Leone and Togo. These countries occupy an area of over 6,140,000 km^2^ and the natural environment in this area consists of subtropical and tropical regions with semi-arid and humid climates [14]. In these communities, traditional herbalists operate closer to the people, taking advantage of the biodiversity of plant species in such areas to cure various diseases and ailments.

**Fig. 1:**
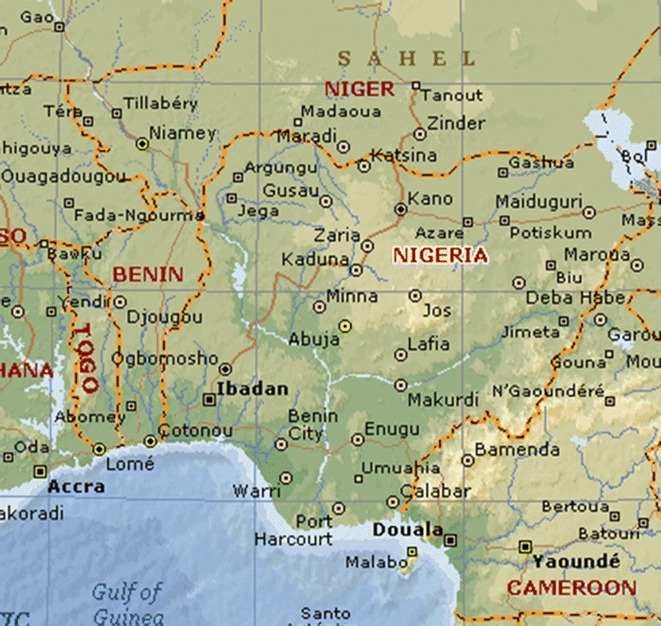
Map showing Nigeria and her neighbours [24]

Nigeria is located in West Africa on the Gulf of Guinea and shares borders with Cameroon (1,690 km) in the East, Chad (87 km) in the Northeast, Niger (1,497 km) in the North, Benin (773 km) in the West and the Atlantic Ocean in the South (Fig. 1) [24]. The country is divided administratively into the Federal Capital Territory (Abuja) and 36 states [25]. As a result of the large surface area occupied by Nigeria, the national territory covers different climatic and ecological zones. Nigeria is rich in biodiversity, with an array of fauna and flora. These include about 20,000 species of insects, almost 1,000 species of birds, 247 species of mammals, 123 species of reptiles, about 1,000 species of fish and about 7,895 species of plants [26]. Consequently ATM is a common and acceptable practice in Nigeria as in many countries in Africa and Asia. Many plants from the Nigerian flora which provide very useful clues for potential therapeutic compounds have been investigated by mostly university research groups. Some of the plants investigated are commonly used in the treatment of several microbial infections [27], a range of neglected tropical diseases including malaria [28], trypanosomiasis [29], as well as diabetes [30] and diverse infections. It is therefore very important to scientifically document the available knowledge for its exploitation towards the enhancement of human health [31].

Several attempts towards the documenting of ethnobotanical and ethnopharmacological knowledge for medicinal plants in Nigeria have been carried out. Raphael has provided a useful work on traditional medicine in Nigeria, about its current status and the future [1], while Soladoye et al. [32] provided a list of 143 plant species belonging to 58 families which were found to be useful for the treatment of haemorrhoids in South Western Nigeria. Idowu et al. [33] also carried out an ethnobotanical survey around Ogun State, South Western Nigeria, in which 38 plant species belonging to 24 families were identified to be used in herbal anti-malarial recipes, while Ajibesin et al. [34] identified a total of 114 medicinal plant species representing 102 genera and 54 families employed in the traditional medical practice of the people of Akwa Ibom State, Nigeria. Adetutu et al. [35] carried out a similar survey of wound-healing plants, while Sonibare and Gbile, listed plants that are being used in the treatment of asthma in South Western Nigeria [17]. Gbolade made an inventory of plants used in the treatment of diabetes in Lagos State and plants used in treating hypertension in Edo State of Nigeria [36, 37]. Idu et al. [38] conducted an ethnobotanical survey of plants among the Esan Tribe of Edo State, and identified 32 plant species belonging to 31 genera and 23 families which have been used for oral healthcare.

One of the goals of our research centre is to document and establish knowledge bases for chemical substances, including those derived from plant use in ATM. Our previous studies have been focused on Central Africa [13, 39–41], including the pharmacokinetics profiling of natural products (NPs) derived from plant materials [42–44]. It has been our aim to extend the study to different regions and eventually to cover the entire continent of Africa. Due to the different works carried out by different research teams on the Nigerian flora, the aim of this survey is to identify those plants used traditionally for the treatment of various ailments in Nigeria that show promising lead-like activity. Our focus has been on the NPs whose measured biological activities correlate with the use of the plant in ATM. In this survey, the ethnobotanical uses of the plant species from Nigeria *versus* the bioactivities of the derived NPs are presented by plant family and in alphabetical order.

## Alliaceae, Amaryllidaceae, Annonaceae, Apocynaceae, Asteraceae and Bignoniaceae

**Fig. 2:**
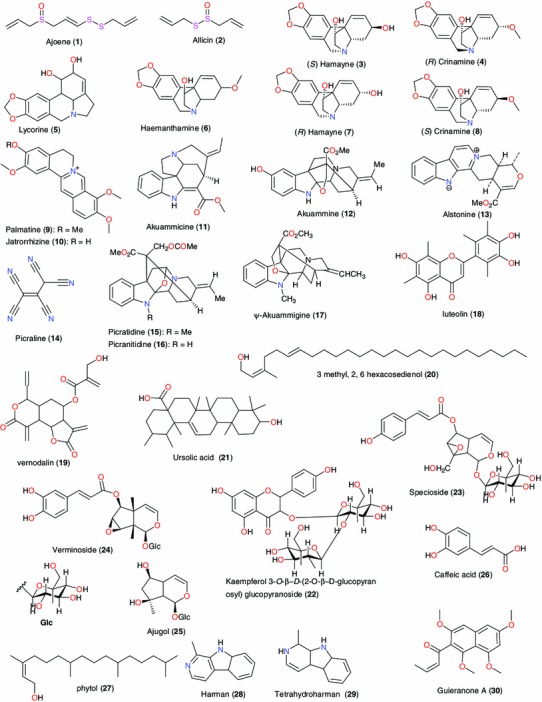
Active principles from *Allium sativum*, *Crinum glaucum*, *Crinum jagus*, *Enantia chlorantha*, *Picralima nitida*, *Struchium sparganophora*, *Spathodea campanulata*, *Guiera senegalensis* and *Morinda lucida*

The ethnobotanical uses of plants in the above families are shown in Table 1, along with the biological activities of the most remarkable isolated NPs. The biological activities which correlate with the enthnobotanical uses of the plants have been highlighted in bold. From the Alliaceae family, ajoene (**1**) and allicin (**2**), Fig. 2, were isolated from the bulb of *Allium sativum* (commonly known as garlic), a plant traditionally used in Nigeria for the treatment of malaria, among diverse uses [45]. Ajeone was active against *Plasmodium berghei* in mice [46], thus validating the ethnobotanical use of the plant, meanwhile allicin was reported as a *P. falciparum* cysteine protease inhibitor [47]. It should be mentioned that rodent malaria is a well-known animal model for testing new compounds and plant extracts. However, trial in humans is decisive to identify a “hit” as “a real hit”; and this is a good way to assess toxicity and safety. *Crinumglaucum* is used in the treatment of cough, asthma, and convulsions in Nigeria [48, 49], while *Crinum jagus* is a plant used in traditional medicine, either singly or in a combination with *Chromoleana odorata* and *Emilia prateramisa* (both of Asteraceae family) in the treatment of all forms of convulsion [50]. Compounds **3**–**8** represent potent alkaloids isolated from *Crinum* sp. (Amaryllidaceae), which exhibit acetylcholinesterase inhibition, the most active alkaloids isolated being hamayne (**7**, IC_50_ = 250 μM) and lycorine (**5**, IC_50_ = 450 μM), while the other alkaloids were comparatively inactive, with haemanthamane (**6**) giving 3 % inhibition and crinamine (**8**) giving 4.4 % inhibition at 50 mg mL^−1^ (174 μM). These results contrast with the positive control physostigmine which gave IC_50_ of 0.25 μM. Thus, cholinesterase activity appears to be associated with the presence of two free hydroxy groups in this structural type of Amaryllidaceae alkaloids. Crinamine has also been isolated from the aerial parts of the Asian subspecies *Crinum asiaticum* var. *japonicum* together with lycorine, norgalanthamine and epinorgalanthamine [51]. The compound showed potent dose dependent inhibition (IC_50_ = 2.7 μM) of HIF-1α in a cell-based reporter gene assay [51]. The other components of the Asian subspecies (from Korea) showed no significant inhibition of HIF-1α induced transcriptional activity.

**Table 1:** Summary of ethnobotanical uses versus measured biological activities of isolated secondary metabolites from; Alliaceae, Amaryllidaceae, Annonaceae, Apocynaceae, Asteraceae and Bignoniaceae plant families

Plant family	Plant name	Use in traditional medicine	Part of plant studied	Active principle	Measured activity	Author and References
Alliaceae	*Allium sativum*	Treatment of **malaria**, seasoning food, cleansing of blood, prevention and fighting of common cold, boost testosterone levels, regulation of blood sugar levels and as an antiseptic	Bulb	**1** and **2**	**Antiplasmodial activity**	Adebayo et al. [45], Perez et al. [46], Coppi et al. [47]
Amaryllidaceae	*Crinum glaucum*	Used in the treatment of cough, asthma, and convulsions. The plant extracts have also exhibited analgesic, anti-inflammatory and antianaphylactic properties	Whole plant	**3**, **4** and **5**	Acetylcholinesterase inhibition	Okpo and Adeyemi, [48] Houghton et al*. [*50]
	*Crinum jagus*	Treatment of all forms of convulsions and some infectious diseases	Whole plant	**5**, **6**, **7** and **8**	Acetylcholinesterase inhibition, HIF-1α inhibition	Azikiwe et al. [49], Houghton et al. [50], Kim et al. [51]
Annonaceae	*Enantia chlorantha*	Treatment of **malaria,** jaundice, dysentery, hypertension, skin, gastric and duodenal ulcers, inflammation, and liver-related diseases and to make unpainted furniture and veneers	Stem bark	**9** and **10**	**Antiplasmodial** and antiviral activities	Adebayo et al. [45], Bhadra and Kumar [52], Bidla et al., [53], Jia et al. [54]
Apocynaceae	*Picralima nitida*	Treatment of **malaria,** diarrhea and as a **painkiller**	Stem bark, seed	**11**, **12**, **13**, **14, 15, 16** and **17**	**Antiplasmodial activity**, antipsychotic and anxiolytic properties and known **potent μ-opioid agonists**	Adebayo et al., [45], Ezeamuzie et al. [55], Okokon et al. [56], Elisabetsky and Costa-Campos [57]
Asteraceae	*Struchium sparganophora*	Treatment of malaria and **measles,** cutaneous, subcutaneous **parasitic infection**, rheumatic pains, **diarrhea, dysentery** as well as **venereal diseases**, as an abortifacient, and in the treatment of inflammatory and **tumor-related** ailments. Also used in the preparation of soup in the South Western part of Nigeria	Leaf	**18**, **19** and **20**	**Antimicrobial** and **antitumour** activities	Kasim et al. [58], Kupchan et al. [59], Liobikas et al. [60], Gnoatto et al. [61]
Bignoniaceae	*Spathodea campanulata*	Extracts of bark, leaves and flowers are used to treat **malaria**, HIV, diabetes mellitus, oedema, dysentery, constipation, gastrointestinal disorders, ulcers, skin diseases, wounds, fever, urethral inflammation, liver complaints and as a poison antidote	Stem bark	**21**	**Antiplasmodial activity**, cardioprotective and aromatase inhibitor	Adebayo et al. [45]
		Treatment of diseases (**ulcers**, dysentery, oedemas, skin eruptions, scabies, wound healing and urethral discharge) and veterinary application have been attributed to the plant in different cultures	Flowers, fruits, leaf and stem bark	**22**, **23**, **24**, **25**, **26** and **27**	**Antioxidant activity**	Elusiyan et al. [62], Picerno et al. [63]

*Enantia chlorantha* (Annonaceae) is an ornamental tree, of up to 30 meters in height, with dense foliage and spreading crown. It’s stem bark is used against fever/malaria by traditional medicine practitioners in the forest regions [44], in addition to its use in the treatment of jaundice, dysentery, hypertension, inflammation, and liver-related diseases [52]. The isolated compounds palmatine (**9**) and jatrorrhizine (**10**) are known to exhibit anti-malarial activity [53], while palmatine also has weak in vitro activity against flavivirus [54]. Seven compounds with anti-malarial properties; akuammicine (**11**), akuammine (**12**), alstonine (**13**), picraline (**14**), picratidine (**15**) picranitidine (**16**) and ψ-akuammigine (**17**) have been isolated from the stem bark and seeds of *Picralima nitida* (Apocynaceae), a plant used in the treatment of malaria and in the management of pains and other ailments [55, 56]. Based on these results, an enterprising Ghanaian hospital started manufacturing standardised 250 mg capsules of the powdered *P. nitida* seed, and sold around the country where they became widely accepted as a safe and effective pain relief product. The extract showed potent and dose-dependent anti-inflammatory, anti-pyretic and anti-malarial activities. Given intraperitoneally, this extract inhibited carrageenan-induced rat paw oedema with IC_50_ of 102 mg kg^−1^, and with the highest dose tested (300 mg kg^−1^) producing 72.2 % inhibition. On the LPS-induced pyrexia in rabbits, 50 mg kg^−1^ of the extract produced a mean percentage antipyrexia of (38.7 %) compared with (29.0 %) by 200 mg kg^−1^ of aspirin. In a 4-day in vivo schizontocidal test in mice infected with *P. berghei*, up to 300 mg kg^−1^ daily for 4 days was ineffective in preventing the development of parasitaemia or the consequent mortality. However, marked inhibitory activity was obtained on multi-drug resistant human *P. falciparium* parasites cultured in vitro. The dose causing 50 % inhibition of parasite growth was 1.75 μg mL^−1^, compared with 0.14 μg mL^−1^ for Chloroquine. The results justify the use of this plant by natives of West Africa in the treatment of malaria. Akuammidine and ψ-akuammigine, are known to be potent μ-opioid agonists, although not particularly selective [57]. Kasim et al. investigated the leaves of *Struchium sparganophora* (Asteraceae), a plant used traditionally to treat malaria and measles [58]. The isolated compounds (**18**–**20**) demonstrated antimicrobial activities against the bacteria *Staphylococcus aureus* (NCTC 6571), *Klebsiella aerogenes* (Welcome Res. Lab.CN 345), *Escherichia coli* (NCTC 9001) and *Proteus vulgaris* (NCTC 8313), as well as against the fungal strains *Candida albicans* (ATCC10231) and *Aspergilus niger* (NCPF3149). Vernodalin (**19**) has been also isolated from *Vernonia amygdalina*, along with vernomygdin, and shown to demonstrate antitumour activities [59]. The anti-malarial activity of ursolic acid (**21**), derived from *Spathodea campanulata* (Bignoniaceae), popularly known as African tulip tree, has been used to endorse the use of the plant in southwestern Nigeria for malaria treatment by drinking the decoction of its stem bark [44]. Ursolic acid also has potential use as a cardioprotective compound [60] and was found to be a weak aromatase inhibitor (IC_50_ = 32 μM) [61]. Additionally, the antioxidant activities of compounds **22**–**27** isolated from the flowers, fruits, leaf and stem bark of the same plant have been investigated by Elusiyan et al. [62]. The results show that the antioxidant principles isolated from the various parts of the plant are verminoside (**24**), from the leaves, stem bark and flowers (EC_50_ = 2.04 μg mL^−1^), specioside (**23**), from the flowers (EC_50_ = 17.44 μg mL^−1^), kampeferol diglucoside (**22**), from the leaves (EC_50_ = 8.87 μg mL^−1^) and caffeic acid (**26**), from the leaves and fruits. Verminoside has also been isolated from *Kigelia Africana*, a plant used in Africa for anti-inflammatory, anti-microbial, and anti-skin-aging effects [63]. The anti-inflammatory activity of the compound has been evaluated by cutaneous irritation in cell cultures and reconstituted human epidermis and revealed significant anti-inflammatory activity. The non antioxidant components isolated in the study include ajugol (**25**), from the stem bark and fruits and phytol (**27**), from the leaves [62]. Alstonine is also known to exhibit antipsychotic and anxiolytic properties [57].

## Combretaceae, Compositae, Connaraceae, Crassulaceae and Ebenaceae

**Fig. 3:**
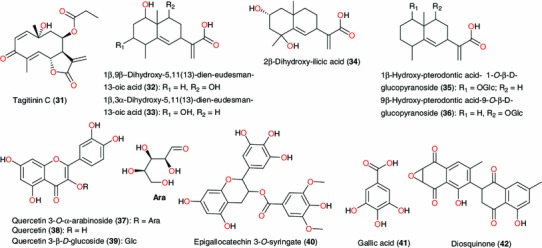
Active principles from *Tithonia diversifola*, *Laggera pterodonta*, *Byrsocarpus coccineus*, *Bryophyllum pinnatum* and *Diospyros mespiliformis*

A summary of the biological activities of the compounds that mark the ethnobotanical uses of the plants of the above families is given in Table 2. *Guiera senegalensis* (Combretaceae) is often used in Nigeria for the treatment of malaria. The leaf extract of the plant harvested in Nigeria showed positive anti-malarial activity in vitro in *Plasmodium yoelii nigeriensis* [64]. The alkaloids harman (**28**) and tetrahydroharman (**29**) and the methoxylated naphthalene derivative guieranone A (**30**), Fig. 3, were shown to be the active principles from this plant species harvested in Mali and São Tomé [65, 66] in addition to its antifungal activity [67]. The leaves of *Tithonia diversifola* (Compositae) have also been extensively used in Nigerian traditional medicine to treat malaria and fevers [45]. Oral decoction of the leaves of *Tithonia diversifolia* are used for treatment of hepatitis, diabetes, malaria, pain, chemoprevention and anti-*Helicobacter pylori* [68–70], external application of dried leaves on wounds and infusion of leaves for the treatment of measles [70]. Goffin et al. [71] isolated the sesquiterpene tagitinin C (**31**) from the leaves of this plant. The anti-plasmodial compound **31** has been proven to be the active principle responsible for the use of the plant leaves in malaria treatment. Tagitinin C also demonstrated therapeutic abilities against gastric ulcer [72]. Another species from the Compositae family, *Laggera pterodonta*, is often used against insect attack, athlete’s foot, skin infections, pediatric malaria and wounds [73]. The plant is also used in the treatment of hepatitis, arthritis, bronchitis and nephritis. Five sesquiterpenes (**32**–**35**), exhibiting antimicrobial activities, have been identified from the species growing in China [74].

**Table 2:** Summary of ethnobotanical uses versus measured biological activities of isolated secondary metabolites from; Combretaceae, Compositae, Connaraceae, Crassulaceae and Ebenaceae plant families

Plant family	Plant name	Use in traditional medicine	Part of plant studied	Active principle	Measured activity	Author and Reference
Combretaceae	*Guiera senegalensis*	Treatment of **malaria**, diarrhea, dysentery, venereal diseases and **microbial infections**	Leaves	**28, 29** and **30**	**Antiplasmodial** and **antifungal** activities	Iwalewa et al. [64], Ancolio et al. [65], Combier et al. [66], Silva and Gomez [67]
Compositae	*Tithonia diversifola*	Treatment of **malaria,** hepatitis, diabetes, malaria, pain, measles, chemoprevention and anti-*Helicobacter pylori*	Leaves	**31**	**Antiplasmodial** and anti-ulcer activities	Kuroda et al. [68], Castillo-Juárez et al. [69], Adebayo et al. [70], Goffin et al. [71], Sánchez-Mendoza et al. [72]
	*Laggera pterodonta*	Against insect attack, **athlete’s foot, skin infections**, pediatric malaria and wounds. Also used in treatment of **hepatitis**, arthritis, bronchitis and nephritis	Air-dried aerial part	**32, 33**, **34**, **35** and **36**	**Antimicrobial Activity**	Egharevba et al. [73]
Connaraceae	*Byrsocarpus coccineus*	Leaf decoction is used for the treatment of venereal diseases and as antidote to arrow poisoning and as remedy for pile, while the decoction of the whole plant is applied to swelling and tumours and also to arrest bleeding, the plant has also been reported as a remedy for diarrhea	Air-dried leaves	**37**, **38** and **39**	Not tested	Ahmadu et al. [75]
Crassulaceae	*Bryophyllum pinnatum*	Treatment of **ulcers**, allergic inflammation and epilepsy	Dried whole plant	**18, 40** and **41**	**Antibacterial activity**	Ogungbamila et al. [76]
Ebenaceae	*Diospyros mespiliformis*	Leaf decoction used for whooping cough treatment and root extracts as worm expellants	Root	**42**	Cytotoxicity	Adeniyi et al. [83]

The leaf extract of *Byrsocarpus coccineus* (Connaraceae) has been used for the treatment of several venereal diseases and as an antidote to arrow poisoning. It also acts as a remedy for piles, while the decoction of the whole plant is applied to swellings and tumours and to arrest bleeding. The plant has also been reported as a remedy for diarrhea. The isolated flavonoid and flavonoid glycosides (**37**–**39**), have been respectively identified as quercetin 3-*O*-*α*-arabinoside, quercetin and quercetin 3-*β*-d-glucoside [75].

*Bryophyllum pinnatum* (Crassulaceae) has diverse uses in ATM. The flavonoid luteolin (**18**), epigallocatechin 3-*O*-syringate (**40**) and gallic acid (**41**) have been identified as the active principles responsible for the antibacterial activity of this plant, which explains why it is used in many West African traditional medicinal recipes for the treatment of ulcers [76]. The main antibacterial constituent was found to be free gallic acid, which accounted for about 0.014 % w/w of the fresh aerial part. However, luteolin and a new acylated flavan-3-ol, epigallocatechin-3-*O*-syringate, were isolated as minor constituents in the active fraction. Luteolin exhibits a wide range of biological activities, including antioxidant activity promotion of carbohydrate metabolism, and immune system modulation. Other in vitro studies suggest luteolin has antiinflammatory activity [77, 78], and that it acts as a monoamine transporter activator [79], a phosphodiesterase inhibitor [80], and an interleukin 6 inhibitor [77]. In vivo studies show that luteolin affects xylazine/ketamine-induced anesthesia in mice [80]. In vitro and in vivo experiments also suggest the compound may inhibit the development of skin cancer [81, 82]. Diosquinone (**42**), derived from the roots of *Diospyros mespiliformis* (Ebenaceae), has shown very good activity against all the cell lines tested with ED_50_ values ranging between 0.18 μg mL^−1^ against Human Glioblastoma (U373) and 4.5 μg mL^−1^ against hormone dependent human prostate cancer (LNCaP) [83]. The relationship with the ethnobotanical use of this plant has not however been established (the leaf decoction has been used for the treatment of whooping cough and root extracts used as worm expellants).

## Euphorbiaceae

**Fig. 4:**
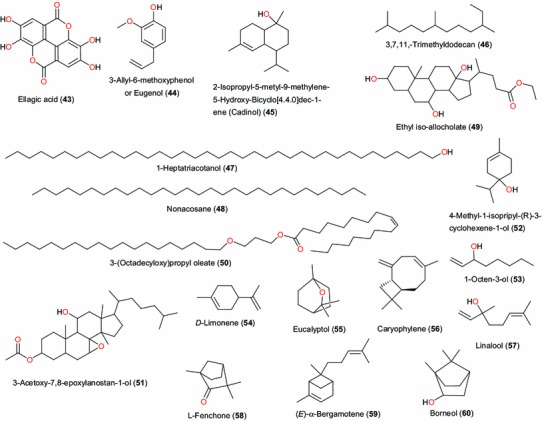
Active principles from *Alchornea cordifolia*—I

Amongst the plants of the Euphorbiaceae family, Tor-Anyiin et al. [84] have reported the use of *Alchornea cordifolia* in the treatment of malaria/fevers in Nigeria (Table 3). Banzouzi et al. [85] identified ellagic acid (**43**), Fig. 4, to be the active ingredient from the stem of this plant, responsible for its anti-malarial activity. The leaves of the same plant are used as a remedy for arthritis, muscle pain and other inflammatory disorders. Okoye et al. carried out an analysis of the volatile oil extracted from the fresh leaves of *A. cordifolia* and revealed the presence of high concentrations of eugenol (**44**: 41.7 %), cadinol (**45**: 2.46 %), caryophylene (**56**: 1.04 %), linalool (**57**: 30.59 %) and (*E*)-*α*-bergamotene (**59**: 4.54 %). These compounds could be contributing to the topical anti-inflammatory effects of *A. floribunda* and *A. cordifolia* leaf extracts [86]. Okoye and Osadebe had also carried out a bioactivity-guided fractionation of the ethyl acetate fraction of the methanol leaf extract of the plant material, leading to the isolation of a new flavonol glycoside, 3,5,7,3′-tetrahydroxyflavone-3-*O*-*α*-*L*-rhamnoside (**61**). The anti-inflammatory activity (50 mg kg^−1^) of this compound was higher than that of the standard anti-inflammatory drug, aspirin (100 mg kg^−1^). The compound also significantly (*p* < 0.001) inhibited heat-induced haemolysis of human erythrocytes in vitro [87]. These results demonstrated that the anti-inflammatory activity of *A. floribunda* leaves may be, in part, a result of the flavonol glycoside, compound **61**.

**Table 3:** Summary of ethnobotanical uses versus measured biological activities of isolated secondary metabolites from Euphorbiaceae plant family

Plant family	Plant name	Use in traditional medicine	Part of plant studied	Active principle	Measured activity	Reference
Euphorbiaceae	*Alchornea cordifolia*	The leaves or leafy stems, as an infusion or chewed fresh, are taken for their sedative, **antimalarial** and antispasmodic activities to treat a variety of respiratory problems including sore throat, cough and bronchitis, genital–urinary problems including venereal diseases and female sterility, and intestinal problems including gastric ulcers, diarrhoea, amoebic dysentery and worms	Stem	**43**	**Antiplasmodial activity**	Tor-Anyiin et al. [84], Banzouzi et al. [85]
		Remedy for arthritis, muscle pain and other acute and chronic **inflammatory disorders**	Leaves	**44**–**51**, **53–60** and **62**	**Anti-inflammatory activity**	Okoye et al. [86]
	*Alchornea floribunda*	Leaves are traditionally used as a remedy for arthritis, muscle pain and other **inflammatory disorders**	Leaves	**61–75**	**Anti-inflammatory activity**	Okoye et al. [86], Okoye and Osadebe [87]
	*Jatropha gossypifolia*	Treatment of various disease conditions such as cough, tuberculosis, bacterial infections and cancerous growths. The leaves of the plant are traditionally being applied to boils, carbuncles, **eczema**, itches, and veneral diseases and also used as febrifuge, while its bark is used as emmenagogue. Seeds are emetic, purgative and used for cancer and body pain. The leaves and seeds are considered as a purgative and are widely used to treat obstinate constipation. Roots are used to treat leprosy, and stem latex possess coagulant activity	Seeds	**76**	**Antifungal** activity	Falodun et al. [88]

**Fig. 5:**
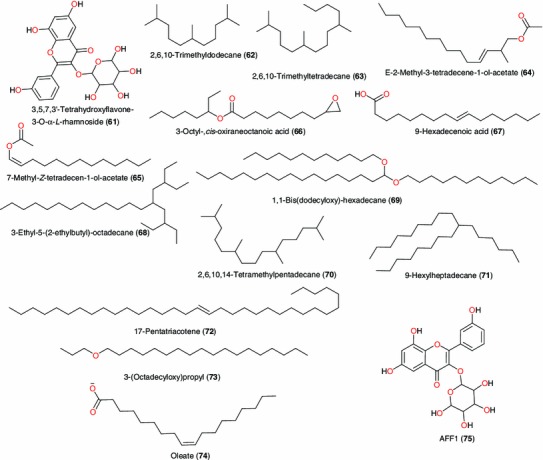
Active principles from *Alchornea floribunda*—II

**Fig. 6:**
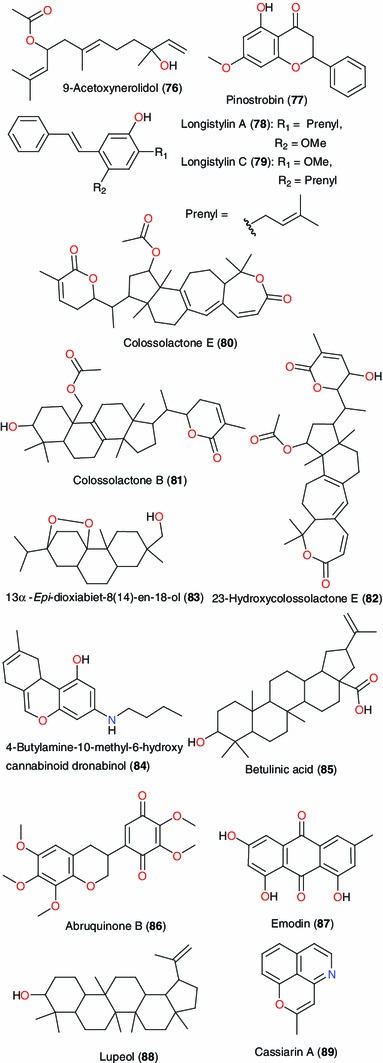
Active principles from *Jatropha gossypifolia*, *Cajanus cajan*, *Ganoderma colossum*, *Hyptis suaveolens*, *Cassia alata*, *Berlina grandiflora*, *Abrus precatorius* and *Cassia siamea*

**Fig. 7:**
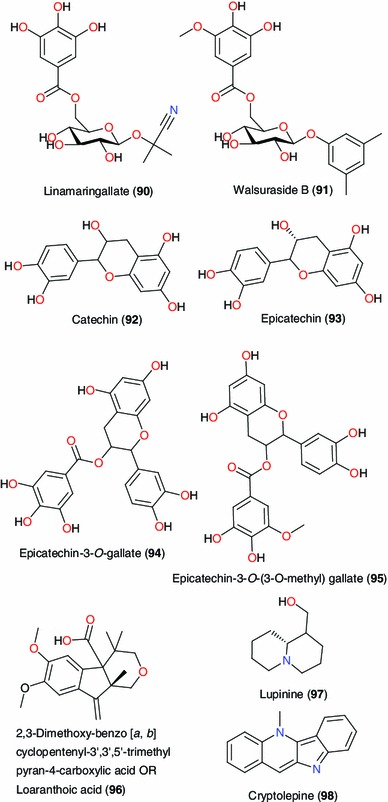
Active principles from *Loranthus micranthus*, *Sida acuta* and *Ixora coccinea*

**Fig. 8:**
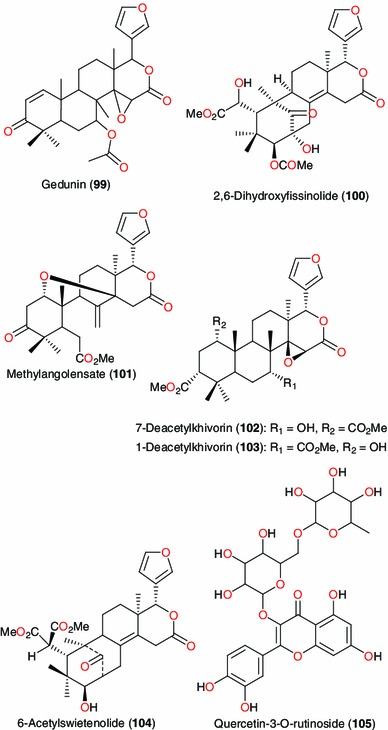
Active principles from *Azadiracta indica*, *Khaya grandifoliola* and *Pavetta crassipes*

**Fig. 9:**
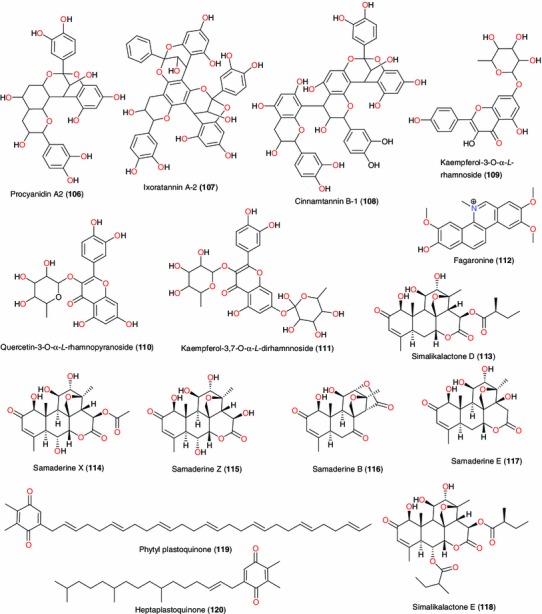
Active principles from *Ixora coccinea*, *Fagara zanthoxyloides*, *Quassia amara*, *Quassia indica* and *Aframomum danielli*

From this same family, *Jatropha gossypifolia* has been used to treat various disease conditions such as cough, tuberculosis, bacterial infections and cancerous growths [88]. The phytochemical studies revealed the presence of some secondary metabolites such as alkaloids, saponins and tannins. There was no activity against the tested bacteria (Gram positive and Gram negative organisms at 2.5–100 mg mL^−1^). The seed extract however showed significant antifungal activity. From the seeds of this plant Falodun et al. carried out a spectroscopic analysis (1D and 2D NMR) of the colourless oil, giving 9-acetoxynerolidol (**76**), which they suggested was the active ingredient [68] Figs. 5–9.

## Fabaceae, Ganodermataceae, Lamiaceae and Loranthaceae

Our survey also involved five species from the Fabaceae or Leguminoceae family, Table 4. These are *Cajanus cajan* used in cancer treatment [89], *Cassia alata* used in the treatment of skin diseases such as ringworm, eczema, pruritis, itching, scabies, ulcers and other related diseases, *Berlina grandiflora* used in the treatment of gastrointestinal disorders, as well as *Abrus precatorius and Cassia siamea*, both used in the treatment of malaria. A dichloromethane (CH_2_Cl_2_) fraction of the leaves of *Cajanus cajan* had IC_50_ value 5–10 μg mL^−1^, with the two constituent stilbenes, longistylins A (**78**) and C (**79**), being primarily responsible, with IC_50_ values of 0.7–14.7 μM against the range of cancer cell lines [89–91].

**Table 4:** Summary of ethnobotanical uses versus measured biological activities of isolated secondary metabolites from; Fabaceae, Ganodermataceae, Lamiaceae and Loranthaceae plant families

Plant family	Plant name	Use in traditional medicine	Part of plant studied	Active principle	Measured activity	Author and Reference
Fabaceae	*Cajanus cajan*	**Cancer** treatment, for the treatment diabetes and as an energy stimulant	Leaves	**77–79**	**Cytotoxicity**	Ashidi et al. [89]
Ganodermataceae (Fungus)	*Ganoderma colossum*	Mushrooms of this genus are known to possess anti-tumour, anti-cancer, **immunomodulatory and immunotherapeutic qualities**	Whole fungus	**80–82**	Antimicrobial activity, **activity against HIV-1 protease**	Ofodile et al. [92], Paterson [93], El Din et al. [94]
Lamiaceae	*Hyptis suaveolens*	Treatment of respiratory tract infections, colds, pain, **fever**, cramps and skin diseases	Leaves	**83**	**Antiplasmodial activity**	Chukwujekwu et al. [95]
Leguminosae	*Cassia alata*	Treatment of skin diseases such as ringworm, eczema, pruritis, itching, scabies, **ulcers** and other related disease	Whole plant	**84**	**Antibacterial activity**	Okwu and Nnamdi [96]
	*Berlina grandiflora*	Treatment of **gastrointestinal disorders**	Whole plant	**85**	**Anthelmitic activity**	Enwerem et al. [97]
	*Abrus precatorius*	Treatment of **malaria**	Stem bark	**86**	**Antiplasmodial activity**	Muhammad et al. [98], Limmatvapirat et al. [99]
	*Cassia siamea*	Treatment of **malaria.** In Asia, stem bark is used as a mild, pleasant, safe purgative; to treat diabetes; a paste is used as a dressing for ringworm and chilblains; the roots are used as an antipyretic; and the leaves are used for the treatment of constipation, **hypertension**, and insomnia	Leaves	**87–89**	**Antiplasmodial activity, vasodialator effect**	Ajaiyeoba et al. [100], Morita et al. [101], Oshimi et al. [102]. Matsumoto et al. [91]
Loranthaceae	*Loranthus micranthus*	Treatment of diarrhea, epilepsy, hypertension and rheumatism	Leafy twigs	**90–95**	Antioxidant activity	Agbo et al. [103]
		Treatment of several diseases including **immune-modifying diseases**	Leaves	**96** and **97**	**Immunostimulatory activity**	Omeje et al. [104]

Ofodile et al. have isolated and characterized three colossolactones; colossolactone E (**80**), colossolactone B (**81**) and 23-hydroxycolossolactone E (**82**), from an *n*-hexane:dichloromethane (2:7) extract of *Ganoderma colossum* [92]. The antimicrobial screening of these compounds revealed that colossolactone E and 23-hydroxycolossolactone E were active against *Bacillus subtilis* and *Pseudomonas syringae*. Potency of the compounds against the tested bacteria supports the use of this mushroom in therapeutic medicine, since this genus of mushrooms is known to possess anti-tumor, anti-cancer, immunomodulatory and immunotherapeutic qualities [93]. Colossolactone B was not active against the bacteria. Other colossolactones (V–VIII and G) and schisanlactone A have also been isolated from the mushroom, *Ganoderma colossum*, harvested from Vietnam, together with colossolactone E (**80**). The isolated compounds were evaluated for inhibition of HIV-1 protease, with IC_50_ values for the most potent compounds ranging from 5 to 13 μg/mL [94]. Colossolactone G showed the most promising anti-viral activity, thus justifying the immunomodulatory and immunotherapeutic qualities of this mushroom.

*Hyptis suaveolens* (Lamiaceae), used in the treatment of respiratory tract infections, colds, pain, fever, cramps and skin diseases has been investigated by Chukwujekwu et al. [95]. A bioactivity-guided fractionation of the petroleum ether extract of the leaves of this plant led to the isolation of the abietane-type diterpenoid endoperoxide, 13*α*-*epi*-dioxiabiet-8(14)-en-18-ol (**83**), a molecule with high anti-plasmodial activity (IC_50_ = 0.1 μg mL^−1^). This might explain the usefulness of the plant for treating fever, which might be due to malaria.

Among the other plants of the Leguminoceae family, the antibacterial activity of 4-butylamine-10-methyl-6-hydroxy cannabinoid dronabinol (**84**), isolated from *Cassia alata*, could explain the use of this plant in the treatment of ulcers, amongst other skin diseases [96]. The anthelmintic activity of betulinic acid (**85**) was also used to validate the use of *Berlina grandiflora* for treating gastrointestinal disorders [97]. The investigations of Muhammad and Amusa have reported the use of the stem bark of *Abrus precatorius* (Leguminosae-Caesalpinioideae) in the treatment of malaria [98]. Limmatvapirat et al. isolated abruquinone B (**86**), which showed anti-plasmodial activity, with IC_50_ = 1.5 μg mL^−1^ against the K1 strain of *P. falciparum*, from the stem bark of this plant [99]. Ajaiyeoba et al. [100] also reported the use of the leaves and stem bark of *Cassia siamea* in the treatment of malaria. Investigation of the leaves of this plant led to the isolation of emodin (**87**), lupeol (**88**) and cassiarin A (**89**), with IC_50_ values of 5.0 μg mL^−1^ against the K1 strain for both compounds **87** and **88** and an IC_50_ value of 0.02 μM for compound **90** [101, 102]. In Asian traditional folk medicine, the stem bark of *Cassia siamea* is used as a mild, pleasant, safe purgative; a decoction of the bark is given to treat diabetes; a paste is used as a dressing for ringworm and chilblains; the roots are used as an antipyretic; and the leaves are used for the treatment of constipation, hypertension, and insomnia [101]. The vasodilator effect of cassiarin A, could explain the use of this plant in the treatment of hypertension, amongst other ailments [102].

From the leafy twigs of *Loranthus micranthus* (Loranthaceae), a parasitic growing on *Hevea brasiliensis*, compounds **90**–**95** have been isolated [103]. The antioxidant activities of the isolated compounds were evaluated using the 2,2-diphenyl-1-picrylhydrazyl (DPPH) assay, giving IC_50_ values varying from 23.8 and 50.1 μM, all being more active than the reference drug chlorogenic acid (with IC_50_ = 67.9 μM) [103]. The immunostimulatory activities of 2,3-dimethoxy-benzo[*a*,*b*]cyclopentenyl-3′,3′,5′-trimethyl pyran-4-carboxylic acid (**96**) and lupinine (**97**) have been used to validate the use of the leaves of this same plant in the treatment of several diseases including immune-modulating diseases [104].

## Malvaceae, Meliaceae, Rubiaceae, Rutaceae and Simaroubaceae

*Sida acuta* (Malvaceae) is widely used for the treatment of malaria in West Africa, among other diseases [105]. The ethanol, aqueous and chloroform extracts are known to display antiplasmodial activity at 3.90, 0.92 and 0.87 μg mL^−1^ respectively against the *P. falciparum* FcM29 strain [106–108]. Banzouzi et al. [107] identified cryptolepine (**98**) as the active ingredient from samples collected in Ivory Coast, West Africa. Cryptolepine analogues have also been isolated from *Cryptolepis sanguinolenta* (Periplocaceae), harvested from diverse locations in West Africa and exhibiting antiplasmodial activity [109–114].

Different parts of *Azadiracta indica* and *Khaya grandifoliola* (Meliaceae) are also used in West Africa for traditional preparations to treat malaria [115–117]. The limonoid gedunin (**99**), isolated from the leaves of *A. indica*, inhibits *P. falciparum* at IC_50_ of 1.25 μg mL^−1^ [115, 118]. Gedunin was also isolated from *K. grandifoliola*, along with the other limonoids 2,6-dihydroxyfissinolide (**100**), methylangolensate (**101**), 7-deacetylkhivorin (**102**), 1-deacetylkhivorin (**103**) and 6-acetylswietenolide (**104**), exhibiting significant antiplasmodial activities [118]. Additionally, gedunin has displayed anticancer activities [119] and Hsp90 inhibition [120].

Among the surveyed plants of the Rubiaceae family, *Pavetta crassipes* has been used in handling respiratory infections and abdominal disorders [121], while the leaves and stems of *Morinda lucida* are used in treating malaria [45, 84] and *Ixora coccinea* is used to treat a variety of infections, including hypertension, menstrual irregularities, sprains, chronic ulcers and skin diseases [122]. A bioactive flavonoid (quercetin-3-*O*-rutinoside, **105**) has been isolated from the aqueous extract of *P. crassipes* leaves, which showed activity against some pathogenic microorganisms, including *Streptococcus pyogenes*, *Corynebacterium ulcerans*, *Klebsiella pneumoniae*, *Neisseria gonorrhoeae*, *Pseudomonas aeruginosa*, and *Escherichia coli* at a concentration < 50 mg mL^−1^ [121]. The compound had minimum inhibitory concentration (MIC) ranging from 6.25 to 12.5 mg mL^−1^ and minimum bactericidal concentration (MBC) ranging from 12.5 to 25 mg mL^−1^. This supports the ethnomedicinal use of the plant in the treatment of respiratory infections and abdominal disorders [121].

The ethanol, CH_2_Cl_2_ and petroleum ether extracts of the leaves and stems of *Morinda lucida* exhibited respective IC_50_ values of 5.70, 5.20 and 3.90 μg mL^−1^ [123]. Cimanga et al. [124] identified ursolic acid (**21**) as the active ingredient, which exhibited antiplasmodial activity at IC_50_ of 3.10 μg mL^−1^ against the chloroquine-sensitive strain of *P. falciparum*. Idowu et al. [122] identified a doubly linked, A-type proanthocyanidin trimer and other constituents of *Ixora coccinea* leaves. The antioxidant and antibacterial properties of the identified compounds (**93**, **106**–**111**) were also investigated. All tested compounds inhibited the growth of *B. subtilis*, while only epicatechin (**93**) and quercetin-3-*O*-*α*-*L*-rhamnopyranoside (**110**) inhibited the growth of *E. coli*. Antioxidant evaluation of isolated compounds revealed that ixoratannin A-2 (**107**) and cinnamtannin B-1 (**108**) were the most active compounds in DPPH, inhibition of lipid peroxidation and nitric oxide radical scavenging assays. This could explain why the plant is effective in the treatment of chronic ulcers. The presence and antiplasmodial property of the alkaloid fagaronine (**112**), with an IC_50_ of 0.018 μM against the 3D7 strain of *P. falciparum*, in *Fagara zanthoxyloides* (Rutaceae), could explain why the roots of this plant are used in preparations against malaria, amongst other applications in ATM [125].

The leaves and stems of plants from the *Quassia* species (Simaroubaceae) are generally employed in anti-malarial preparations, *Q. amara* (also called bitterwood tree) having the highest anti-malarial reputation for curative and preventive purposes in the Simaroubaceae family [45, 126]. Simalikalactone D (**113**), samaderine X (**114**), samaderine Z (**115**), samaderine B (**116**) and samaderine E (**117**) have been isolated from *Q. amara* and *Q. indica*, displaying anti-plasmodial properties in the range 0.010 and 0.210 μg mL^−1^ [127, 128]. Simalikalactone E (**118**), was also isolated from French Guianian *Quassia amara*. The compound displayed anti-malarial activity and was shown to be less toxic than simalikalactone D (**113**) [129].

Odukoya et al. [130] have shown that the alcohol and petrol extracts of seeds of *Aframomum danielli* (Zingiberaceae), used traditionally as a food spice and also as an anti-inflammatory agent, inhibit the soya 5-lipoxygenase enzyme and thus may show anti-inflammatory activity. Compounds isolated from the active extract were shown to be long chain polypropenyl benzoquinone derivatives. Phytyl plastoquinone (**119**) and heptaplastoquinone (**120**) were respectively isolated from the petrol and alcohol extracts, both compounds inhibiting 5-lipoxygenase at 6.25 and 18.5 μM, respectively, compared with the standard drug Fisetin, which inhibited the enzyme at 0.92 μM, Table 5.

**Table 5:** Summary of ethnobotanical uses versus measured biological activities of isolated secondary metabolites from; Malvaceae, Meliaceae, Rubiaceae, Rutaceae and Simaroubaceae plant families

Plant family	Plant name	Use in traditional medicine	Part of plant studied	Active principle	Measured activity	Author and Reference
Malvaceae	*Sida acuta*	Treatment of **malaria,** ulcer, fever, gonorrhea, abortion, breast cancer following inflammation, wound infections	Stem	**98**	**Antiplasmodial** activity	Obute [105], Bertani et al. [106], Banzouzi et al. [107], Karou et al. [108]
Meliaceae	*Azadiracta indica*	Treatment of **malaria.** It is also known for anthelmintic, antifungal, antidiabetic, antibacterial, antiviral, contraceptive and sedative properties	Leaves	**99**	**Antiplasmodial** activity	Isah et al. [115]
	*Khaya grandifoliola*	Treatment of **malaria**, as remedy against rheumatoid arthritis. Extracts also show anti-inflammatory and toxic effects	Bark, roots and seeds	**99–104**	**Antiplasmodial** and anticancer activities and Hsp90 inhibition	Agbenanusi et al. [116, 117], Bickii et al. [118], Kamath et al. [119], Brandt et al. [120]
Rubiaceae	*Pavetta crassipes*	Management of **respiratory infections** and abdominal disorders	Fresh plant	**105**	**Antimicrobial** activity	Bello et al. [121]
	*Morinda lucida*	Treatment of **malaria**, diabetes,	Leaves, stem bark	**20**	**Antiplasmodial** activity	Awe and Makinde [123], Cimanga et al. [124]
	*Ixora coccinea*	Treatment of a variety of infections; hypertension, menstrual irregularities, sprains, **chronic ulcers** and skin diseases	Leaves	**93**, **106–111**	**Antioxidant** activities	Idowu et al. [122]
Rutaceae	*Fagara zanthoxyloides*	Treatment of **malaria.** The stem and the root of the plant are both used as chewing stick in Nigeria particularly among the Yoruba ethnic group in the South-Western part of the country	Root	**112**	**Antiplasmodial activity**	Odebiyi and Sofowora [125]
Simaroubaceae	*Quassia amara*	Treatment of **malaria,** as a digestive, treat fever, against hair parasites (lice, fleas), and Mosquito larvae in ponds (and do not harm the fishes). Extracts of *Quassia* wood or bark act as a natural insecticide	Leaves, stem	**113**	**Antiplasmodial activity**	Ajaiyeoba et al. [126]
	*Quassia indica*	Treatment of **malaria**, rheumatism, asthma, skin diseases and as an insecticide, especially against white ants	Leaves, stem	**114**–**118**	**Antiplasmodial activity**	Bertani et al. [127] Kitagawa et al. [128], Cachet et al. [129]
Zingiberaceae	*Aframomum danielli*	Used as traditional food spice, as an anti-inflammatory agent and also for crop protection	Seeds	**119** and **120**	Lipoxygenase inhibition	Odukoya et al*.* [130]

## Conclusions

The results presented in this review represent an overview of the biological activities of selected NPs isolated from plants used in traditional medicine in Nigeria. Our intention has been to focus on those plants whose ethnobotanical uses correlate with the biological activities of the derived NPs. Even though this report does not claim to be exhaustive, the goal of documenting the baseline knowledge, from which further investigations could be carried out, has been achieved. This work has also led to the collection of a chemical database for plants used in traditional medicine in Nigeria (Naijaplant), to be made available separately. The plant sources, geographical collection sites, chemical structures of pure compounds as well as their spectroscopic data, were retrieved from literature sources comprising data collected from 176 articles from 68 peer reviewed journals, spanning the period 1971 to 2013. Our survey consisted in collecting data from the literature sources, mainly from MSc and PhD theses from Nigerian University libraries and also using the author queries in major natural product and medicinal chemistry journals. The collected data includes plant sources, uses of plant material in traditional medicine, plant families, region of collection of plant material, isolated metabolites and type (e.g. flavonoid, terpenoid, etc.), measured biological activities of isolated compounds, and any comments on significance of isolated metabolites on the chemotaxonomic classification of the plant species (as commented in the literature). This survey suggests that information gathered from compounds derived from African medicinal plants, in particular from Nigeria, could be a suitable starting point for extensive drug discovery projects, especially for some of the most frequently mentioned diseases like malaria, since malaria is the most endemic disease in Africa and plants used in the treatment of malaria have been the most investigated on the continent [39, 40, 45, 131–134].
